# Prevalence of and factors associated with atypical presentation in bacteremic urinary tract infection

**DOI:** 10.1038/s41598-022-09222-9

**Published:** 2022-03-25

**Authors:** Junpei Komagamine, Taku Yabuki, Daichi Noritomi, Taro Okabe

**Affiliations:** 1 Department of Internal Medicine, National Hospital Organization Tochigi Medical Center, 1-10-37, Nakatomatsuri, Utsunomiya, Tochigi, Japan; 2 Department of Internal Medicine, Saiseikai Utsunomiya Hospital, 911-1, Takebayashimachi, Utsunomiya, Tochigi, Japan

**Keywords:** Bacterial infection, Urinary tract infection, Urological manifestations

## Abstract

A delay in the diagnosis of urinary tract infection (UTI) is not uncommon. Atypical presentation is often cited as one of the causes of diagnostic delays. However, few studies have investigated the prevalence of atypical presentation and determined factors associated with atypical presentation at initial contact among patients with UTI. Therefore, a retrospective and prospective cohort study using chart review was conducted in two acute care hospitals. We included 285 consecutive patients hospitalized for bacteremic UTI. The primary outcome was atypical presentation, defined as the absence of any urinary tract symptom or sign at initial contact. Of all patients, the median age was 82 years, 186 (65.3%) were women, and 53 (18.6%) had dementia. Urinary tract symptoms and signs were absent at initial contact in 144 patients (50.5%; 95% CI 44.7–56.4%). The multivariable analysis revealed that older age, male sex, dementia, and early visit from symptom onset were significantly associated with an increased risk of atypical presentation. Patients with atypical presentation were less likely to receive a correct diagnosis at initial contact than patients with urinary tract symptoms and signs (OR 0.30; 95% CI 0.17–0.51). Atypical presentation in patients with bacteremic UTI is common and negatively affects the correct diagnosis of UTI.

## Introduction

Urinary tract infection (UTI) is one of the most common infections requiring hospitalization^1^ and an important cause of sepsis^2^. Sepsis mortality due to UTI is reported to be 10–30%^2,3^. Given that a delay in appropriate antimicrobial therapy is associated with poor patient outcomes^4,5^, it is important to make a correct diagnosis early and initiate appropriate antimicrobial therapy for UTI.

Nonetheless, a delay in the diagnosis of UTI is not uncommon^6^. Atypical presentation is often cited as one of the causes of diagnostic delays^6,7^ because typical UTI symptoms and signs, such as dysuria, frequency and costovertebral angle tenderness, are often lacking in patients with delayed diagnosis of UTI. Based on past and recent studies, one of the risk factors associated with the absence of urinary tract symptoms and signs in patients with UTIs is advanced age^6,8–10^. However, the aim of these past studies^6,8–10^ was to investigate not the risk factors for the absence of urinary tract symptoms and signs among patients with UTIs but the clinical features of elderly patients with UTIs. Therefore, few studies have investigated the prevalence of and risk factors for atypical presentation among patients with UTIs. Moreover, no studies have ever been conducted to investigate the effect of atypical presentation on diagnostic or treatment delays for UTI. Thus, we conducted a multicenter observational study to determine the prevalence of and risk factors associated with atypical presentation and investigate the effect of atypical presentation on a correct diagnosis in patients with UTI.

## Results

A total of 285 patients with bacteremic UTI were included. The baseline characteristics of the included patients at initial presentation are shown in Table 1 and Table S1. Of the 285 patients, the median age was 82 (IQR 73–87) years, 186 (65.3%) were women, 53 (18.6%) had dementia, 26 (9.1%) had benign prostatic hypertrophy, and 102 (35.8%) had diabetes. The median time to initial presentation from symptom onset was one day (0–2). The most common symptom at presentation was fever or chills (n = 233, 81.8%), followed by altered mental status (n = 93, 32.6%) and weakness (n = 86, 30.2%). On physical examination at initial presentation, lower abdominal tenderness and costovertebral angle tenderness were present in 24 (8.4%) and 91 (31.9%) patients, respectively. Among all the cases, 82 (28.8%) and 169 (59.3%) were cases of complicated UTI and pyelonephritis, respectively. The most common pathogen was *Escherichia coli* (n = 211, 74.0%), followed by *Klebsiella* species (n = 27, 9.5%).

**Table 1:** Baseline characteristics of 285 patients with bacteremic urinary tract infection.

Characteristics^a^	Total (n = 285)	Atypical presentation	
		Yes (n = 144)	No (n = 141)
Median age (IQR)	82 (73–87)	83 (78–82)	80 (67–86)
Women	186 (65.3)	86 (59.7)	100 (70.9)
Nursing home	44 (15.4)	25 (17.4)	19 (13.5)
**The time to initial visit from symptom onset**			
Median days (IQR) Less than 24 h	1 (0–2) 114 (40.0)	1 (0–2) 70 (48.6)	1 (0–2) 44 (31.2)
**Medical history**			
Hypertension Dementia Stroke Ischemic heart disease Benign prostatic hypertrophy Diabetes mellitus	156 (54.7) 53 (18.6) 62 (21.8) 24 (8.4) 26 (9.1) 102 (35.8)	85 (59.0) 37 (25.7) 33 (22.9) 9 (6.3) 14 (9.7) 54 (37.5)	71 (50.4) 16 (11.4) 29 (20.6) 15 (10.6) 12 (8.5) 48 (34.0)
**Symptoms at presentation**			
Fever Nausea or vomiting Weakness Flank pain Back pain Dysuria Frequent urination Dyspnea Lower abdominal pain Altered mental status	233 (81.8) 78 (27.4) 86 (30.2) 11 (3.9) 37 (13.0) 16 (5.6) 36 (12.6) 26 (9.1) 14 (4.9) 93 (32.6)	112 (77.8) 35 (24.3) 51 (35.4) 0 (0.0) 0 (0.0) 0 (0.0) 0 (0.0) 13 (9.0) 0 (0.0) 54 (37.5)	121 (85.8) 43 (30.5) 35 (24.8) 11 (7.8) 37 (26.2) 16 (11.4) 36 (25.5) 13 (9.2) 14 (9.9) 39 (27.7)
**Physical signs at presentation**			
Lower abdominal tenderness Costovertebral angle tenderness	24 (8.4) 91 (31.9)	0 (0.0) 0 (0.0)	24 (17.0) 91 (64.5)
**Initial diagnosis at first contact**			
Urinary tract infection or urosepsis Fever (unspecified) Pneumonia	202 (70.9) 27 (9.5) 14 (4.9)	85 (59.0) 22 (15.3) 12 (8.3)	117 (83.0) 5 (3.6) 2 (1.4)
In-hospital mortality	17 (6.0)	11 (7.6)	6 (4.3)

^a^Values are expressed as numbers with the percentages of the total numbers, unless otherwise stated.

For the primary outcome, any urinary tract symptom or sign was absent at initial presentation in 144 (50.5%) patients (Table 2). For the secondary outcomes, the proportion of patients without fever and any urinary tract symptoms or signs was 11.2%, and the proportion of patients with a correct UTI diagnosis at initial presentation was 70.9%. Antibiotic therapy was started at initial contact for any reason in 223 (78.3%). The most common initial diagnosis at first contact was UTI or urosepsis (n = 202, 70.9%), followed by unspecified fever (n = 27, 9.5%) and pneumonia (n = 14, 4.9%).

**Table 2:** Primary and secondary outcomes^a^.

	Total (n = 285)
**Primary outcome**	
No urinary tract symptoms or signs^b^	144 (50.5)
**Secondary outcomes**	
No fever and no urinary tract symptoms or signs^b^	32 (11.2)
A correct UTI diagnosis at initial contact	202 (70.9)
Initiation of antibiotic therapy at initial contact	223 (78.3)

^a^Values are expressed as numbers with the percentages of the total numbers, unless otherwise stated.

^b^Urinary tract symptoms include dysuria, urgency, frequency, suprapubic pain, flank pain, back pain, costovertebral angle tenderness or lower abdominal tenderness on physical examination at the time of the initial presentation.

UTI, urinary tract infection.

In the multivariable analysis, older age (OR 2.16; 95% CI 1.22–3.83), male sex (OR 1.85; 95% CI 1.09–3.13), dementia (OR 2.19; 95% CI 1.11–4.30), and early visit from symptom onset (OR 1.77; 95% CI 1.07–0.2.95) were predictive factors for atypical presentation in UTI patients (Table 3). Patients without urinary tract symptoms or signs were less likely to be diagnosed with UTI correctly at presentation (OR 0.30; 95% CI 0.17–0.51) and to be prescribed antimicrobial drugs at initial contact (OR 0.33; 95% CI 0.18–0.61) than patients with any urinary tract symptoms or signs.

**Table 3:** Results of the multivariable analysis^a^ for factors associated with no urinary symptoms or signs at presentation among patients with bacteremic urinary tract infections.

Variables	Odds ratios (95% CI)	*P* value
Aged 75 years or older	2.16 (1.22–3.83)	0.008
Male sex	1.85 (1.09–3.13)	0.02
Dementia	2.19 (1.11–4.30)	0.006
Ischemic heart disease	0.43 (0.17–1.06)	0.07
Initial visit from symptom onset < 24 h	1.77 (1.07–2.95)	0.03

^a^Variables were removed one-by-one using a backward stepwise method until all remaining variables had a p value of less than 0.2. The following variables were considered: age, sex, hypertension, dementia, stroke, ischemic heart disease, benign prostatic hypertrophy, diabetes, and time to initial visit from symptom onset. The level of statistical significance was set at 5%

CI, confidence interval.

## Discussion

Our findings showed that half of the patients with bacteremic UTI had no urinary tract symptoms or signs at initial presentation. Independent predictive factors for the absence of urinary tract symptoms and signs were older age, male sex, dementia, and early visit from symptom onset. Moreover, the absence of urinary tract symptoms and signs at initial presentation was associated with an increased risk of a diagnostic delay of UTI and initiation of antimicrobial therapy.

Our results are consistent with those of past studies^6,10–12^ reporting that more than half of patients with UTIs had no urinary tract symptoms. However, the proportion of correct diagnoses for bacteremic UTI at initial contact in the present study was 70.9%, while that of a past study conducted more than two decades was 43%^6^. This implies that the diagnostic accuracy for bacteremic UTI at initial contact has been improved in the past two decades. Nonetheless, a substantial proportion of bacteremic UTI patients were not diagnosed correctly at initial presentation. Therefore, some strategies to improve the accuracy of bacteremic UTI diagnosis at initial presentation are needed.

In the present study, advanced age and dementia were independent predictive factors for the absence of urinary tract symptoms and signs. This result supports past^6,8,10^ and recent studies^9^ reporting that UTI patients who were older were less likely to have urinary tract symptoms and signs. In addition, our research revealed that the absence of urinary tract symptoms and signs at initial presentation was associated with an incorrect initial diagnosis for UTI and delayed initiation of antimicrobial therapy. Given that elderly UTI patients are more likely to die than younger UTI patients^9^ and that diagnostic and treatment delays may result in the poor prognosis of UTI patients^4,5^, some efforts to diagnose UTI correctly among elderly patients with dementia will be warranted.

Male sex was another independent predictive factor for the absence of urinary tract symptoms and signs in our research. Given that UTI has been more frequently studied in women than in men^9^, it is no surprise that the presenting features of men with UTI are atypical. It may be similar to ischemic heart disease, for which symptoms in women with angina are atypical because classical symptoms of angina are mainly based on male patients^13^. Infection/inflammation of the male accessory gland is difficult to diagnose based on just the clinical history and physical examination^14^. Therefore, it is possible that atypical presentation of bacteremic UTI is more frequent in men. Our findings indicate that it is difficult to differentiate UTI from other diseases in men based on only clinical symptoms and signs because urinary tract symptoms and signs are often absent. Moreover, given the high prevalence of asymptomatic bacteriuria among elderly men^15–18^, an early correct diagnosis of UTI may be difficult, even if urine laboratory tests are performed. Given that there are few studies investigating the clinical features of men with UTI^9^, further studies are warranted to investigate the clinical features of men with UTI and develop strategies to diagnose UTI in men.

### Strength and weakness

This study was the first to investigate risk factors for atypical presentation and the effect of atypical presentation on a diagnostic delay in bacteremic UTI patients. To avoid the incorrect inclusion of infectious and noninfectious diseases other than UTI, we used a strict definition of UTI as a reference standard. Nonetheless, several limitations should be mentioned. First, the data collected in a real-world practice were used by using electronic medical records. Therefore, the information analyzed in the present study might not be accurate. Moreover, the inclusion of only two hospitals in the same area limits the generalizability of our findings. Second, lower abdominal tenderness and costovertebral angle tenderness were not documented or evaluated in some patients. Therefore, the presence of these physical signs was underestimated in the present study. However, given that physicians who suspect UTI generally perform these examinations, it is possible that no documentation reflects atypical presentation in UTI patients. Third, there might have been a substantial loss of targeted UTI patients due to the lack of submission of blood or urine cultures by principal physicians. Fourth, it is uncertain that our results can be generalized to UTIs without bacteremia, which is more common than UTIs with bacteremia. Fifth, acute prostatitis might be incorrectly included in the present study because tenderness of the prostatic gland was not evaluated in all cases. Sixth, overemphasis on avoidance of a delayed diagnosis for bacteremic UTI might increase the overuse of tests and overdiagnosis of UTI, which might result in the overuse of antimicrobial drugs^11,19^. Finally, the majority of patients included in the present study were more than 75 years old. Therefore, this limits the generalizability of our results and may cause bias because some of independent variables used in the multivariable regression analysis were related to age. Nonetheless, past studies have also reported that most patients with bacteremic UTI were elderly^4,10^.

## Methods

### Study setting and design

A retrospective and prospective multicenter cohort study was conducted by using medical electronic records to determine the prevalence of and risk factors associated with atypical presentation in UTI patients. National Hospital Organization Tochigi Medical Center and Saiseikai Utsunomiya Hospital are located in Utsunomiya, Japan. These are the two largest general community hospitals in the region, providing acute care for a population of approximately 0.5 million. This study was approved by the Medical Ethical Committee of National Hospital Organization Tochigi Medical Center and the Medical Ethical Committee of Saiseikai Utsunomiya Hospital. This research was conducted in accordance with the Ethical Guidelines for Epidemiological Research in Japan and was carried out in accordance with the Declaration of Helsinki. The need for individual informed consent was formally waived by the Medical Ethical Committee of National Hospital Organization Tochigi Medical Center and the Medical Ethical Committee of Saiseikai Utsunomiya Hospital because data were collected without contacting the patients. This research was retrospectively registered at the University Hospital Medical Network Clinical Trials Registry (UMIN-CTR) on September 26, 2020 (No. UMIN000041899).

### Inclusion and exclusion criteria

All consecutive patients who were hospitalized due to UTI in Tochigi Medical Center (from September 2014 to March 2021) and Saiseikai Utsunomiya Hospital (from January 2019 to December 2020) were included. We included only UTI patients who had bacteremia and satisfied the definition of UTI. An accurate diagnosis of UTI is difficult in clinical practice. Moreover, the clinical distinction between asymptomatic bacteriuria and UTI is often difficult^15^. Therefore, to avoid the inclusion of infectious diseases other than UTI and noninfectious diseases, we included UTI patients with the same bacterial pathogen isolated from both urine and blood. Patients hospitalized due to renal abscess, proctitis, and cystitis were excluded.

Based on past studies^20,21^ and guidelines^22^, bacteremic UTI was defined if all of the following were satisfied: (1) the patient experienced fever or chills, any symptoms of cystitis (dysuria, urgency, frequency, suprapubic pain, or lower abdominal tenderness on physical examination), or any symptoms of pyelonephritis (flank pain, back pain, or costovertebral angle tenderness on physical examination) during the index episode; (2) the same bacterial pathogen was isolated in concurrent urinary and blood cultures, and there were no other sources of infection; and (3) the bacterial pathogen was present at ≥ 10^4^ CFU/ml of urine. UTI with any indwelling catheter, neurogenic bladder, obstructive uropathy, or urinary retention due to benign prostatic hypertrophy was defined as a complicated bacteremic UTI. During the study period, a total of 285 patients who met the inclusion criteria were included in the final analysis.

### Data collection and outcome measures

Physicians reviewed electronic medical records and retrieved information on patient age, sex, past medical history, medication use, time to initial hospital visit from symptom onset, symptoms, physical findings, initial diagnosis at the first visit, and prognosis. Chronic symptoms were not collected.

The primary outcome was the proportion of patients with atypical presentation at initial contact. Atypical presentation was defined as the absence of any new urinary tract symptoms and signs. Urinary tract symptoms and signs were defined as dysuria, urgency, frequency, suprapubic pain, flank pain, back pain, or lower abdominal tenderness or costovertebral angle tenderness on physical examination. The secondary outcome was the proportion of patients with the absence of new urinary tract symptoms and signs and no fever at initial contact. Fever was defined as a body temperature of 38.0 °C of greater at any site. Other secondary outcomes were the proportion of patients who were diagnosed correctly as having UTI at the initial visit and the proportion of patients who were prescribed any antimicrobial drugs at the initial visit.

### Statistical analysis

Descriptive statistics were used to report the baseline characteristics of the study population. To determine the predictive factors associated with atypical presentation at initial contact, multivariable analysis with binary logistic regression was performed. The associations between the primary outcome and selected variables were examined. The following variables were considered: age, sex, hypertension, dementia, stroke, ischemic heart disease, benign prostatic hypertrophy, diabetes, and time to initial visit from symptom onset. The variables were removed one-by-one in the backward stepwise method until all remaining variables had a p value of less than 0.2. To investigate the hypothesis that atypical presentation can result in diagnostic and treatment delays for UTI, we also investigated the association between the absence of urinary tract symptoms and signs and a correct diagnosis of UTI and initiation of antimicrobial therapy at initial presentation. The level of statistical significance was set at 5%. These analyses were performed by using Stata version 15 (LightStone, Tokyo, Japan).

## Conclusions

The absence of urinary tract symptoms and signs at initial presentation was common and associated with an increased risk of a delay in both diagnosis and therapy in patients with bacteremic UTI. Independent predictive factors for the absence of urinary tract symptoms and signs were older age, male sex, dementia, and early visit from symptom onset. Given that diagnostic and treatment delays may result in the poor prognosis of UTI patients, further studies are warranted to investigate risk factors associated with the absence of urinary tract symptoms and signs and develop strategies for a correct diagnosis in UTI patients without urinary tract symptoms and signs.

## Supplementary Information

Supplementary Information.

## Data Availability

All data generated during this study are included in this published article and its Supplementary Information files.
