# Supplementary material for: Prevalence of and factors associated with atypical presentation in bacteremic urinary tract infection
Source: Sci Rep. 2022 Mar 25;12:5197. doi: 10.1038/s41598-022-09222-9 (PMC8956699; doi:10.1038/s41598-022-09222-9)
Supplement: Supplementary file 1 — Supplementary Information. [file 41598_2022_9222_MOESM1_ESM.pdf]

Table S1. Baseline characteristics of 285 patients with bacteremic urinary tract infection.

Characteristics[a]	Total (n = 285)	Atypical presentation[b]	
		Yes (n = 144)	No (n = 141)
Age			
Median (IQR)	82 (73–87)	83 (78–82)	80 (67-86)
75 years or older	205 (71.9)	117 (81.3)	88 (62.4)
Ambulance use	202 (70.9)	110 (76.4)	92 (65.3)
Use of antibiotics before presentation	19 (6.7)	10 (6.9)	9 (6.4)
Vital signs at presentation, median (IQR)			
Body temperature	38.4 (37.6–39.2)	38.5 (37.6-39.1)	38.4 (37.4-39.4)
Systolic blood pressure	129 (107–149)	127 (104–149)	130 (115–149)
Diastolic blood pressure	68 (58–80)	66 (56–80)	70 (59–80)
Heart rate	102 (88–116)	102 (89–116)	102 (87–115)
Respiratory rate	23 (18-28)	23 (18–28)	23 (19–28)
Laboratory tests			
Serum glucose[b]	137 (110–191)	137 (112–192)	136 (109-191)
Hemoglobin A1c[b]	6.3 (5.6–7.4)	6.4 (5.6–7.5)	6.2 (5.6–7.3)
Positive nitrates on dipsticks[b]	98 (57.0)	42 (51.2)	56 (62.2)
Positive leukocyte esterase on dipsticks[b]	244 (88.4)	124 (87.9)	120 (88.9)
Physicians caring for patients			
Resident	90 (31.6)	45 (31.2)	45 (31.9)
Attending doctor	195 (68.4)	99 (68.8)	96 (68.1)
Causative bacteria			
Escherichia coli	211 (74.0)	108 (75.0)	103 (73.1)
Klebsiella species	27 (9.5)	13 (9.0)	14 (9.9)
Enterococcus faecalis	9 (3.2)	4 (2.8)	5 (3.6)
Citrobacter species	9 (3.2)	5 (3.5)	4 (2.8)
Proteus species	8 (2.8)	5 (3.5)	3 (2.1)
Pseudomonas. aeruginosa	7 (2.5)	2 (1.4)	5 (3.6)
Final diagnosis			
Complicated UTI	82 (28.8)	41 (28.5)	41 (29.1)
Pyelonephritis	169 (59.3)	59 (41.0)	110 (78.0)
Causative factors			
Urinary catheter-related	23 (8.1)	13 (9.0)	10 (7.1)
Neurological bladder	18 (6.3)	12 (8.3)	6 (4.3)

Obstruction due to prostate hypertrophy	10 (3.5)	7 (4.9)	3 (2.1)
Urinary stone-related	40 (14.0)	14 (9.7)	26 (18.4)
Uremia-associated	0 (0.0)	0 (0.0)	0 (0.0)
Duration of hospital stay, median days (IQR)	14 (10–21)	15 (10-26)	13 (9–18)

[a]Values are expressed as numbers with the percentages of the total numbers, unless otherwise stated.

[b]Data were documented or evaluated in 258, 167, 172, and 276 patients for serum glucose, hemoglobin A1c, nitrates on dipsticks, and leukocyte esterase on dipsticks, respectively.

IQR, interquartile range; UTI, urinary tract infection.
